# Detection method for reverse transcription recombinase-aided amplification of avian influenza virus subtypes H5, H7, and H9

**DOI:** 10.1186/s12917-024-04040-9

**Published:** 2024-05-16

**Authors:** Zongshu Zhang, Zichuang Zhang, Chunguang Wang, Xianghe Zhai, Wenjing Wang, Xi Chen, Tie Zhang

**Affiliations:** 1 College of Veterinary Medicine, Hebei Agricultural University, Baoding, China; 2 Institute of Special Animal and Plant Sciences, Chinese Academy of Agricultural Sciences, Changchun, China

**Keywords:** Avian influenza virus, Reverse transcription recombinase-aided amplification, Lateral flow dipstick, Real-time fluorescence

## Abstract

**Background:**

Avian influenza virus (AIV) not only causes huge economic losses to the poultry industry, but also threatens human health. Reverse transcription recombinase-aided amplification (RT-RAA) is a novel isothermal nucleic acid amplification technology. This study aimed to improve the detection efficiency of H5, H7, and H9 subtypes of AIV and detect the disease in time. This study established RT-RAA-LFD and real-time fluorescence RT-RAA (RF-RT-RAA) detection methods, which combined RT-RAA with lateral flow dipstick (LFD) and exo probe respectively, while primers and probes were designed based on the reaction principle of RT-RAA.

**Results:**

The results showed that RT-RAA-LFD could specifically amplify H5, H7, and H9 subtypes of AIV at 37 °C, 18 min, 39 °C, 20 min, and 38 °C, 18 min, respectively. The sensitivity of all three subtypes for RT-RAA-LFD was 10^2^ copies/µL, which was 10 ∼100 times higher than that of reverse transcription polymerase chain reaction (RT-PCR) agarose electrophoresis method. RF-RT-RAA could specifically amplify H5, H7, and H9 subtypes of AIV at 40 °C, 20 min, 38 °C, 16 min, and 39 °C, 17 min, respectively. The sensitivity of all three subtypes for RF-RT-RAA was 10^1^ copies/µL, which was consistent with the results of real-time fluorescence quantification RT-PCR, and 100 ∼1000 times higher than that of RT-PCR-agarose electrophoresis method. The total coincidence rate of the two methods and RT-PCR-agarose electrophoresis in the detection of clinical samples was higher than 95%.

**Conclusions:**

RT-RAA-LFD and RF-RT-RAA were successfully established in this experiment, with quick response, simple operation, strong specificity, high sensitivity, good repeatability, and stability. They are suitable for the early and rapid diagnosis of Avian influenza and they have positive significance for the prevention, control of the disease, and public health safety.

**Supplementary Information:**

The online version contains supplementary material available at 10.1186/s12917-024-04040-9.

## Background

Avian influenza (AI), caused by avian influenza virus (AIV), is an acute, highly contagious disease, which can also infect humans [1, 2]. AIV can be divided into highly pathogenic avian influenza viruses and low pathogenic avian influenza viruses [3]. Highly pathogenic avian influenza causes high mortality in poultry, which was classified as a notifiable disease by the World Organization for Animal Health (WOAH) and a class I infectious disease by China [4, 5]. AIV has a wide range of hosts, not only infecting a variety of animals, for example, birds, but also jumping across species barriers to infect humans [6–8]. Human deaths could be caused by AIV infections, which has been reported in several influenza pandemics. There are a large number of AIV subtypes with high variability, but, no cross-immunity have been found between them, which brought frequent outbreaks of epidemics. At present, highly pathogenic strains are only found in some H5 and H7 subtypes of AIV [9]. Although the H9 subtype of AIV is a low pathogenic virus, it has a wide epidemic scope and high isolation rate. The H9 subtype is easy to recombine with other subtypes, which brings difficulties to the prevention and control work [10]. Therefore, rapid detection of H5, H7, and H9 subtypes of AIV is crucial in controlling the epidemic in the poultry industry and safeguarding human health.

There are a variety of detection methods for AIV. Although the commonly used detection method for virus isolation is relatively accurate, it is cumbersome to operate and it has high requirements for the culture conditions [11, 12]. The commonly used molecular diagnostic technology is reverse transcription polymerase chain reaction (RT-PCR), of which real-time fluorescence quantification RT-PCR (RFQ-RT-PCR) has high sensitivity. However, since RT-PCR relies on a complex thermal cycle process, it has defects such as high requirements for the professional level of instruments and operators and long detection time [13], which limits the application in rapid detection. To avoid the thermal cycling process in RT-PCR, some isothermal amplification technologies have been developed and gradually applied to nucleic acid amplification detection. Among them, the reaction temperature for nucleic acid sequence-based amplification is 42 °C, which can directly amplify RNA, but there are problems such as insufficient sensitivity and false positives in the detection of some viruses [14, 15]. Rolling circle amplification is an isothermal amplification technology based on the rolling circle specific replication method of circular DNA molecules in nature [16]. The reaction temperature is 37 °C, and SNP analysis can be performed, but the required template must be single-stranded circular DNA. If the sample does not meet the requirements, it will need annealing and cyclization treatment, and the reaction time will take longer. Loop-mediated isothermal amplification is a constant temperature nucleic acid amplification technology proposed by Notomi and his team in 2000 [17]. It can react at 60–65 °C with strong specificity and high amplification efficiency, but the primer design is more complicated, 3 ∼4 pairs of primers need to be designed for 6 ∼8 different regions [18]. Recombinase polymerase amplification (RPA) and Recombinase-aided amplification (RAA) are isothermal amplification methods that have been developed in recent years. The principle of both methods is similar, but the recombinase of RPA is usually derived from T4 phage, while the recombinase of RAA is derived from bacteria or fungi, which have a wider variety of sources [19, 20].

RecA recombinase from E. coli, single-stranded DNA binding protein, and DNA polymerase, were applied in RAA [21]. This process of in vitro DNA amplification does not require high temperature. Generally, the same target fragment can be obtained at 37 °C-42 °C for 15–30 min, which is the same as the traditional high temperature polymerase chain reaction (PCR), the detection was completed by agarose electrophoresis, lateral flow dipstick (LFD) and real-time monitoring [22, 23]. This method has been widely used in the detection of a variety of pathogens, such as Carp edema virus [24], Mycoplasma pneumoniae [25], Leishmania [26], pseudorabies virus [19], and Vibrio parahaemolyticus [27].

In this study, a pair of specific primers and probes were designed for the conserved region of the hemagglutinin (HA) gene sequence of H5, H7, and H9 subtypes of AIV. The detection method of reverse transcription recombinase-aided amplification (RT-RAA) combined with LFD (RT-RAA-LFD) and real-time fluorescence RT-RAA (RF-RT-RAA) detection method combined with exo probe were established, respectively. The two detection methods with high sensitivity and strong specificity based on RT-RAA, avoid the complex thermal cycle process of RT-PCR and RFQ-RT-PCR. This study aims to provide two new methods for the detection of H5, H7, and H9 subtypes of AIV and technical support for the effective prevention and control of the disease.

## Results

### Optimization of reaction conditions for RT-RAA-LFD method

**Fig. 1:**
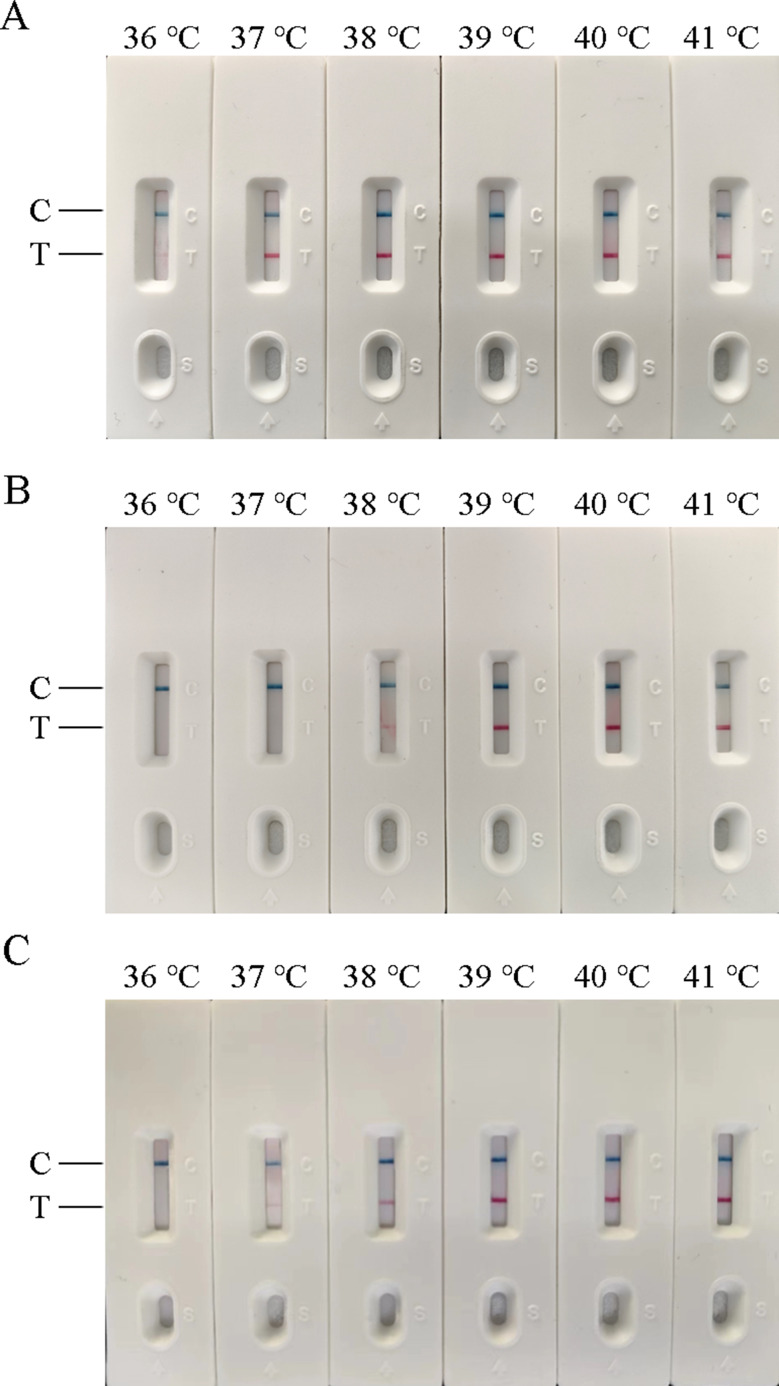
Optimization of reaction temperature for RT-RAA-LFD method. (**A**), (**B**), and (**C**) were the optimized results of the reaction temperature of H5, H7, and H9 subtypes of AIV RT-RAA-LFD method, respectively. In (**A**), clear detection lines were seen at 37–41 °C; in (**B**), clear detection lines were seen at 39–41 °C; in (**C**), clear detection lines were seen at 38–41 °C. According to the optimal reaction conditions, the one with the lowest required reaction temperature and a clear detection line was selected as the optimal temperature, 37 °C for AIV-H5, 39 °C for AIV-H7, and 38 °C for AIV-H9

**Fig. 2:**
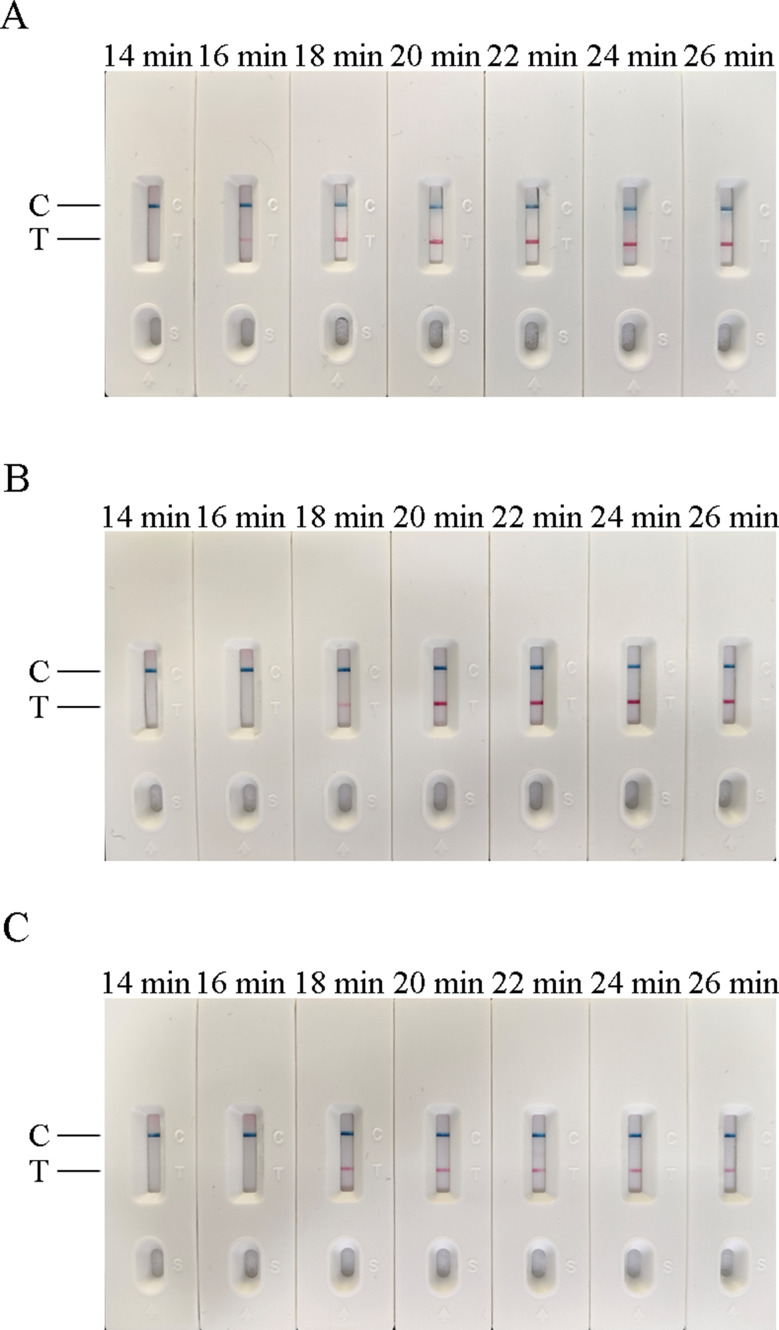
Optimization of reaction time of RT-RAA-LFD method. (**A**), (**B**), and (**C**) were the optimized results of the reaction time of H5, H7, and H9 subtypes of AIV RT-RAA-LFD method, respectively. In (**A**) and (**C**) clear detection lines were visible at 18–26 min; in (**B**) the detection line was visible at 18–26 min, but the detection line at 18 min was not particularly clear, and the clearest detection line appeared around 20 min. According to the optimal reaction condition with the shortest required reaction time and clear detection line, 18 min was selected as the optimal time for AIV-H5 and AIV-H9, and 20 min was selected as the optimal time for AIV-H7

**Fig. 3:**
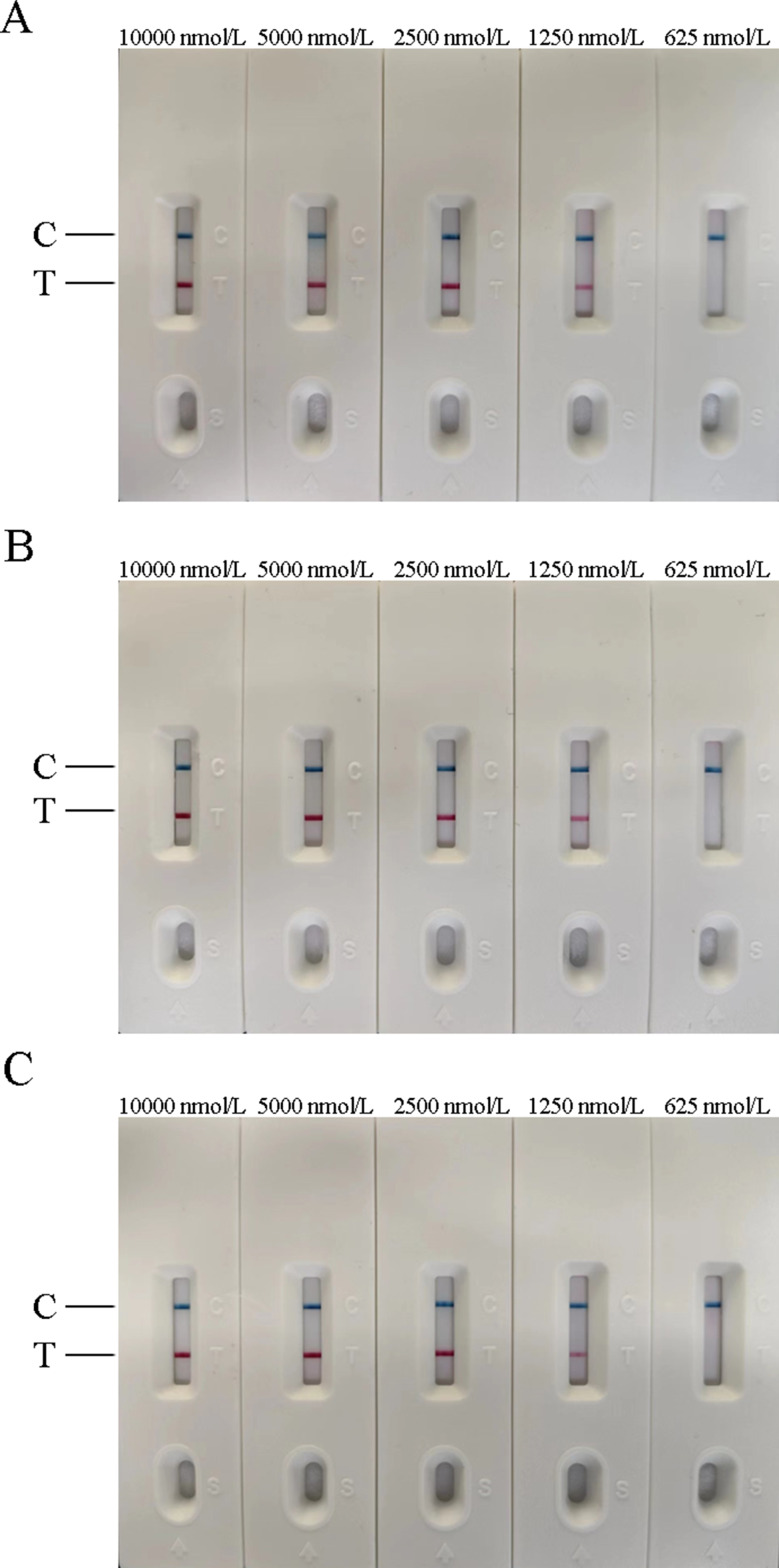
Optimization of primer and probe concentrations for RT-RAA-LFD method. (**A**), (**B**), and (**C**) were the optimized results of primer and probe concentrations for H5, H7, and H9 subtypes of AIV RT-RAA-LFD method, respectively. In (**A**), (**B**), and (**C**), the detection lines were seen when the concentrations of primer and probe were 1250 nmol/L, and the color of the detection lines became more obvious with the rise of the concentration. The required concentration of 1250 nmol/L was lower and the material loss was less. Therefore, 1250 nmol/L was selected as the optimal concentration

In this experiment, the temperature, time, and concentration of primer and probe in the AIV-H5, AIV-H7, and AIV-H9 RT-RAA-LFD reaction systems were screened respectively. According to the judgment criteria of the optimal reaction conditions, the optimal time and temperature for AIV-H5, AIV-H7, and AIV-H9 detection were 18 min at 37 °C, 20 min at 39 °C, and 18 min at 38 °C, respectively. The optimal concentrations of primers and probes of the three subtypes for detection were 1250 nmol/L (Figs. 1, 2 and 3).

### Optimization of reaction conditions for RF-RT-RAA method

**Fig. 4:**
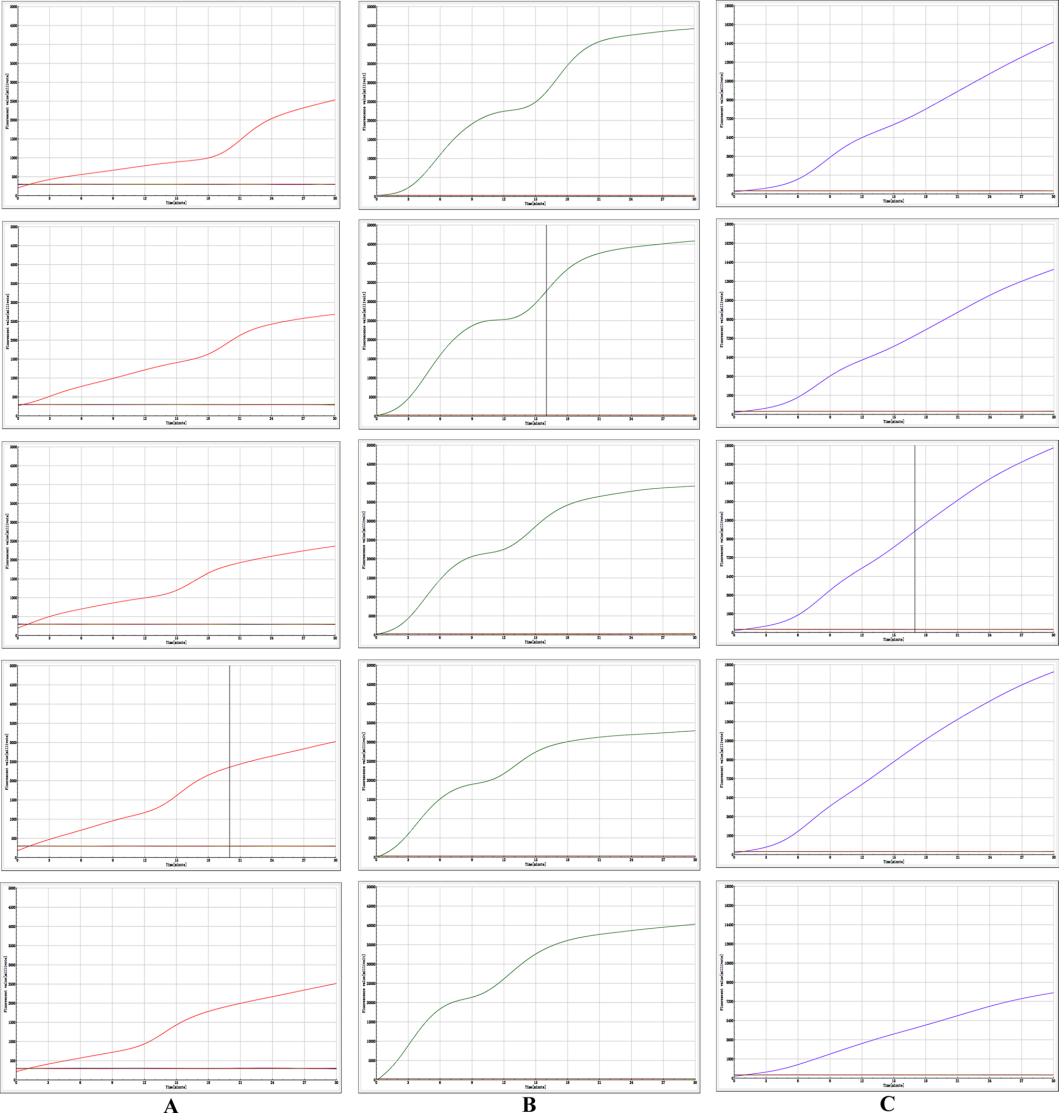
Optimization of reaction conditions for RF-RT-RAA method. (**A**), (**B**), and (**C**) were the optimized reaction conditions of H5, H7, and H9 subtypes of AIV RF-RT-RAA method, respectively, which were 37 °C, 38 °C, 39 °C, 40 °C, and 41 °C from top to bottom. After a comprehensive analysis of fluorescence signal peak value and amplification efficiency, the optimal conditions were 40 °C, 20 min for AIV-H5, 38 °C, 16 min for AIV-H7, and 39 °C, 17 min for AIV-H9

In this experiment, the reaction temperature and time for AIV-H5, AIV-H7, and AIV-H9 were optimized, respectively. By observing the fluorescence signal value of the amplification curve and analyzing the slope of the amplification curve, it was concluded that the fluorescence value of AIV-H5 was the highest at 40 °C and the reaction efficiency was the best for 20 min; the fluorescence value of AIV-H7 was the highest at 38 °C and the reaction efficiency was the best for 16 min; the fluorescence value of AIV-H9 was the highest at 39 °C and the reaction efficiency was the best for 17 min (Fig. 4). According to the standard of optimal reaction conditions, the optimal reaction temperature and time for AIV-H5, AIV-H7, and AIV-H9 detection were 40 °C, 20 min, 38 °C, 16 min, and 39 °C, 17 min, respectively.

### Specificity of RT-RAA-LFD and RF-RT-RAA methods

**Fig. 5:**
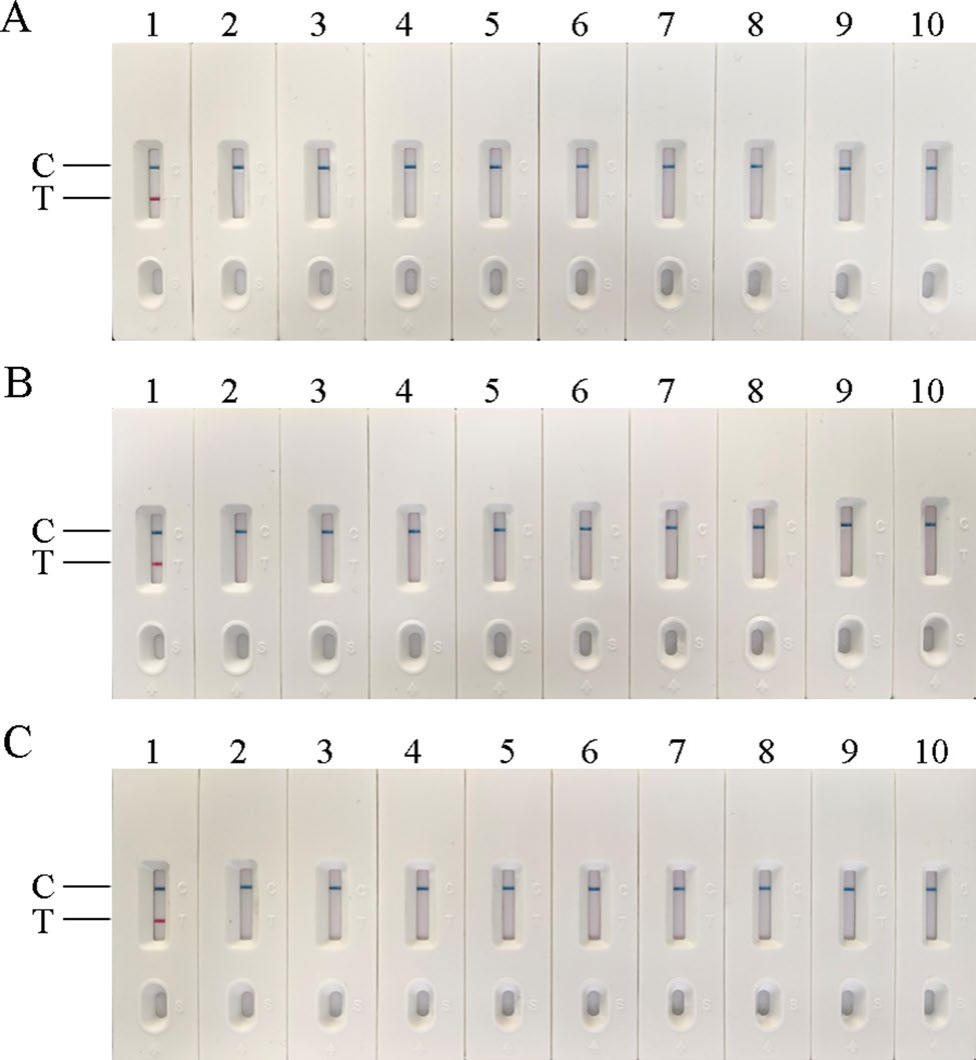
Specificity of RT-RAA-LFD method. (**A**), (**B**), and (**C**) were the specificity test results of H5, H7, and H9 subtypes of AIV RT-RAA-LFD method, respectively. In (**A**), 1: AIV-H5, 2: AIV-H7, 3: AIV-H9; In (**B**), 1: AIV-H7, 2: AIV-H5, 3: AIV-H9; In (**C**), 1: AIV-H9, 2: AIV-H5, 3: AIV-H7; In (**A**), (**B**), and (**C**), 4–10 were AIV-H1N1, AIV-H3N2, Influenza B viruses (Victoria), Influenza B viruses (Yamagata), NDV, IBV, and ILTV, respectively. In (**A**), (**B**), and (**C**), only obvious red detection lines were observed on the corresponding test strips of AIV-H5, AIV-H7, and AIV-H9, respectively, while other corresponding test strips showed negative results

**Fig. 6:**
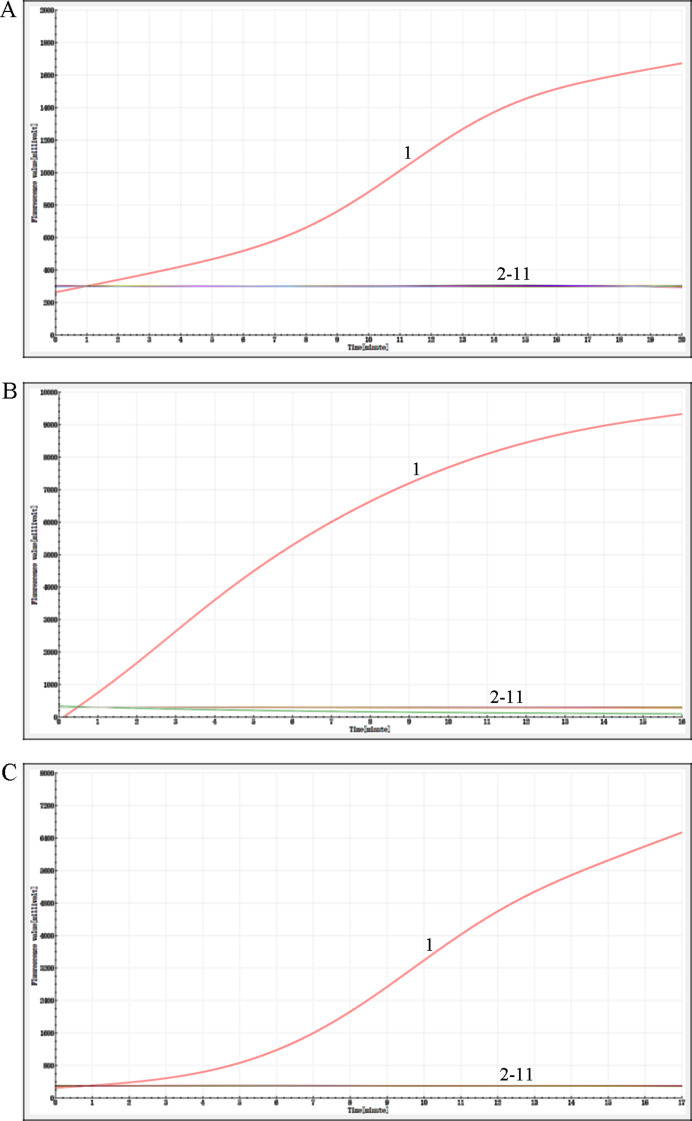
Specificity of RF-RT-RAA method. (**A**), (**B**), and (**C**) were the specificity results of H5, H7, and H9 subtypes of AIV RF-RT-RAA method, respectively. In (**A**), 1: AIV-H5, 2: AIV-H7, 3: AIV-H9; In (**B**), 1: AIV-H7, 2: AIV-H5, 3: AIV-H9; In (**C**), 1: AIV-H9, 2: AIV-H5, 3: AIV-H7; In (**A**), (**B**), and (**C**), 4–11 were AIV-H1N1, AIV-H3N2, Influenza B viruses (Victoria), Influenza B viruses (Yamagata), NDV, IBV, ILTV, and negative control, respectively. Only AIV-H5, AIV-H7, and AIV-H9 showed obvious amplification curves in (**A**), (**B**), and (**C**), respectively, while no amplification curves appeared in other pathogens and negative controls

The results showed that both RT-RAA-LFD and RF-RT-RAA were positive only in the primers and probes corresponding to the H5, H7, and H9 subtypes of AIV, on the contrary, all other groups were negative (Figs. 5 and 6). It could be proved that the primers and probes of H5, H7, and H9 subtypes of AIV designed in this study had high specificity and had no cross-reaction with other similar disease pathogens.

### Sensitivity of four methods of detection

**Fig. 7:**
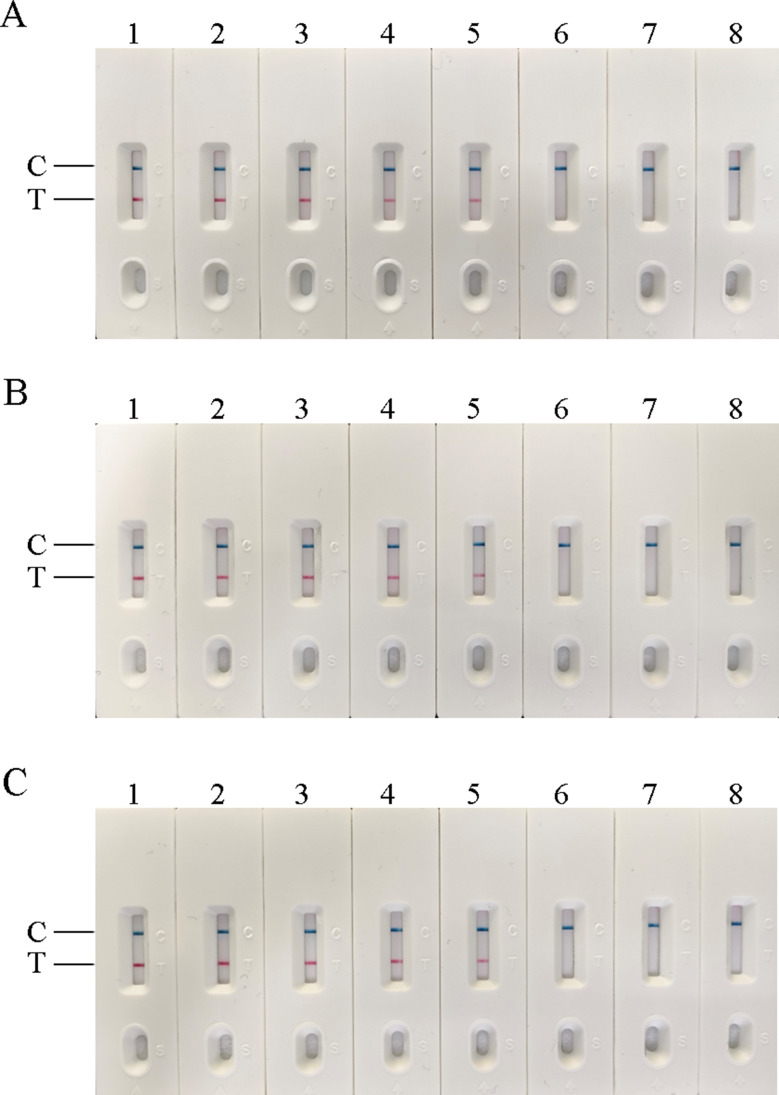
Sensitivity of RT-RAA-LFD. (**A**), (**B**), and (**C**) were the sensitivity results of RT-RAA-LFD for H5, H7, and H9 subtypes of AIV, respectively. 1–7 were plasmids of 10^6^ copies/µL to 10^0^ copies/µL, respectively; 8 was the negative control. In (**A**), (**B**), and (**C**), when the template concentration was 10^2^ copies/µL, the detection lines were obvious, so the MDL of AIV-H5, AIV-H7, AIV-H9 RT-RAA-LFD method was all 10^2^ copies/µL

**Fig. 8:**
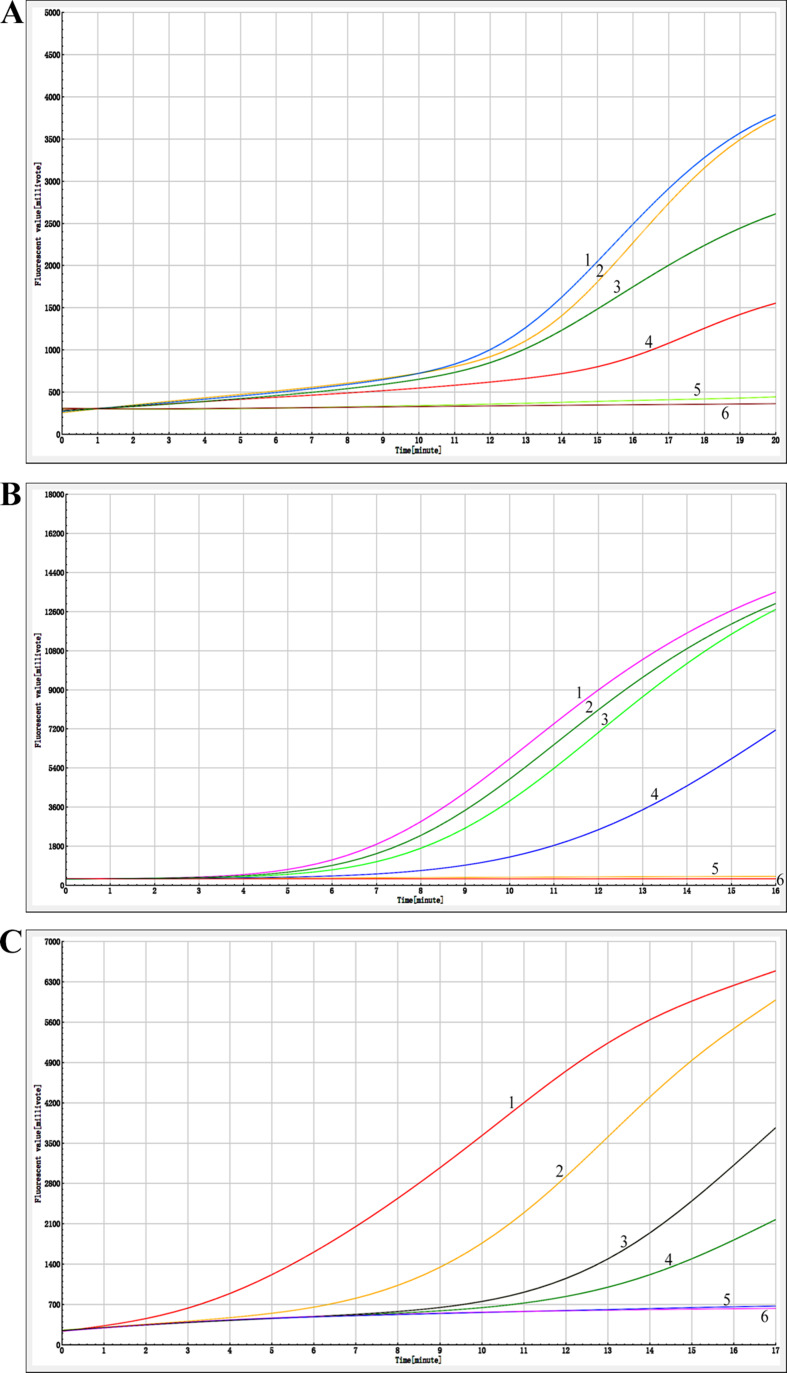
Sensitivity of RF-RT-RAA. (**A**), (**B**), and (**C**) were the sensitivity results of RF-RT-RAA for H5, H7, and H9 subtypes of AIV, respectively. 1–5 were plasmids of 10^4^ copies/µL to 10^0^ copies/µL, respectively; 6 was the negative control. In (**A**), (**B**), and (**C**), when the template concentration was 10^1^ copies/µL, obvious amplification curves were still visible, so the MDL of AIV-H5, AIV-H7, AIV-H9 RF-RT-RAA method was all 10^1^ copies/µL

**Fig. 9:**
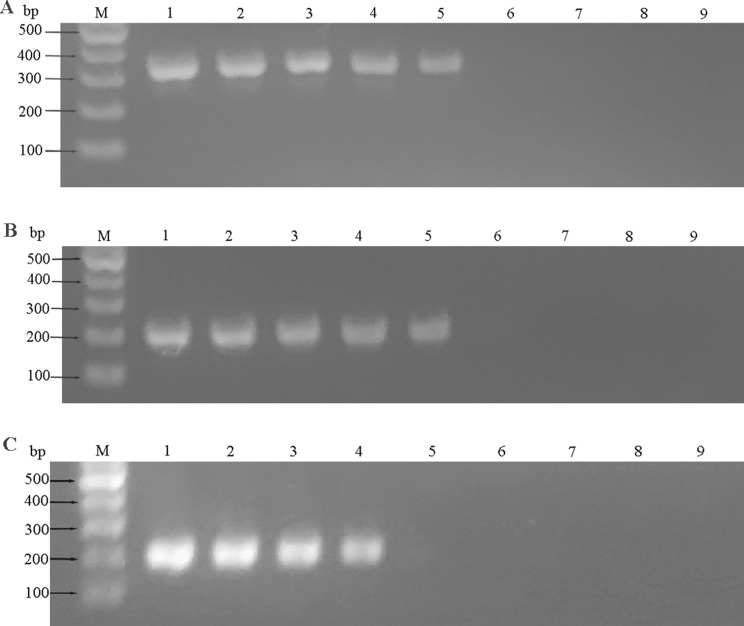
Sensitivity of RT-PCR-agarose electrophoresis method. (**A**), (**B**), and (**C**) were the sensitivity results of RT-PCR-agarose electrophoresis for H5, H7, and H9 subtypes of AIV, respectively. (**A**), (**B**), and (**C**) were all cropped gel images, corresponding to Supplementary Fig. 1, Supplementary Fig. 2, and Supplementary Fig. 3 in the Supplementary Information file, respectively, which showed the full-length gels. 1–8 were plasmids of 10^7^ copies/µL to 10^0^ copies/µL, respectively; 9 was the negative control. The template concentration in (**A**) and (**B**) was 10^3^ copies/µL and the template concentration in (**C**) was 10^4^ copies/µL, clear target bands could be seen. When the template concentrations were lower, no target bands could be seen, so the MDL of AIV-H5 and AIV-H7 RT-PCR-agarose electrophoresis was 10^3^ copies/µL, the MDL of AIV-H9 RT-PCR-agarose electrophoresis was 10^4^ copies/µL

**Fig. 10:**
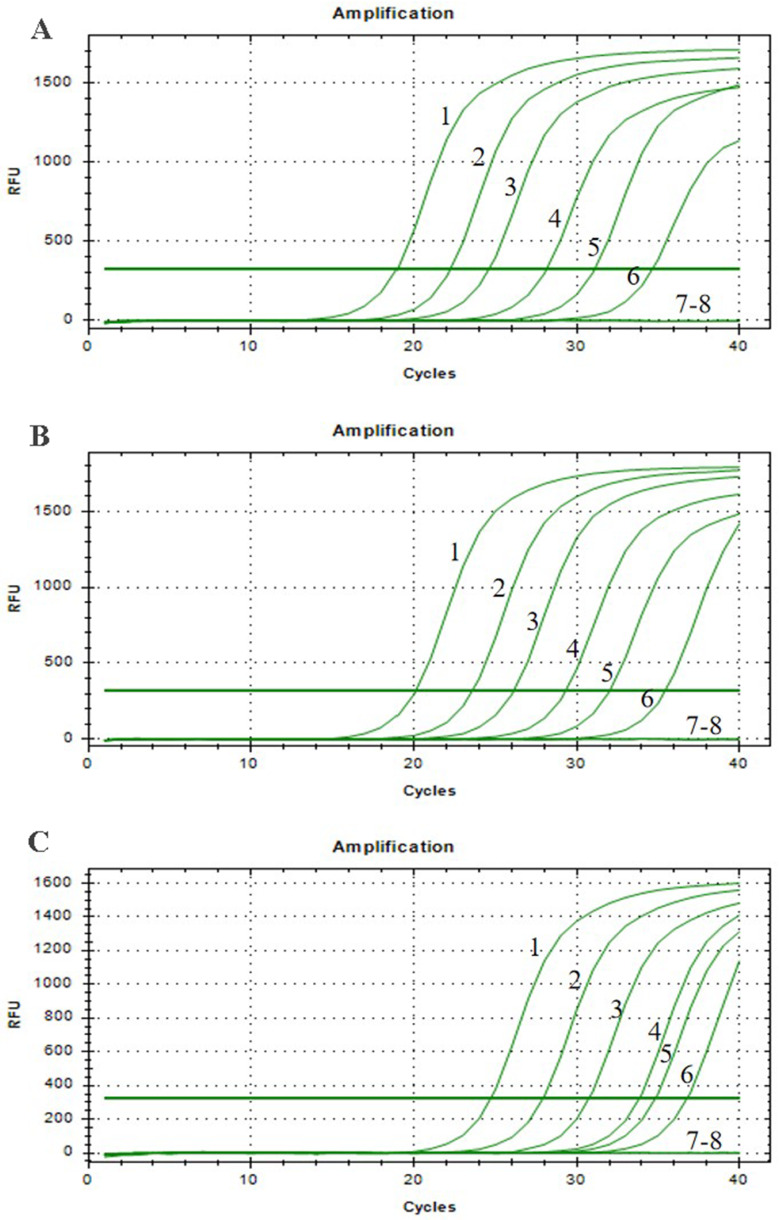
Sensitivity of RFQ-RT-PCR. (**A**), (**B**), and (**C**) were the sensitivity results of RFQ-RT-PCR for H5, H7, and H9 subtypes of AIV, respectively. 1–7 were plasmids of 10^6^ copies/µL to 10^0^ copies/µL, respectively; 8 was the negative control. In (**A**), (**B**), and (**C**), when the template concentration was 10^1^ copies /µL, the amplification curves were still obvious, so the MDL of AIV-H5, AIV-H7, and AIV-H9 in RFQ-RT-PCR was all 10^1^ copies/µL

AIV-H5, AIV-H7, and AIV-H9 were detected by RT-RAA-LFD, RF-RT-RAA, RT-PCR-agarose electrophoresis, and RFQ-RT-PCR, respectively. The minimum detection limit (MDL) of all three viruses by RT-RAA-LFD method was 10^2^ copies/µL (Fig. 7); the MDL of all three viruses by RF-RT-RAA method could reach 10^1^ copies /µL (Fig. 8); the results of RT-PCR-agarose electrophoresis showed that the MDL of AIV-H5 and AIV-H7 was 10^3^ copies/µL, and the MDL of AIV-H9 was 10^4^ copies/µL (Fig. 9). RFQ-RT-PCR was used to detect those three viruses with a minimum of 10^1^ copies/µL (Fig. 10). Above results showed that the sensitivity of RT-RAA-LFD was slightly lower than RFQ-RT-PCR, which was 10 ∼100 times higher than that of RT-PCR-agarose electrophoresis. The sensitivity of RF-RT-RAA was consistent with RFQ-RT-PCR, which was 100 ∼1000 times higher than that of RT-PCR-agarose electrophoresis. It proved that RT-RAA-LFD and RF-RT-RAA established in this study had high sensitivity.

### Repeatability and stability of RT-RAA-LFD and RF-RT-RAA methods

**Fig. 11:**
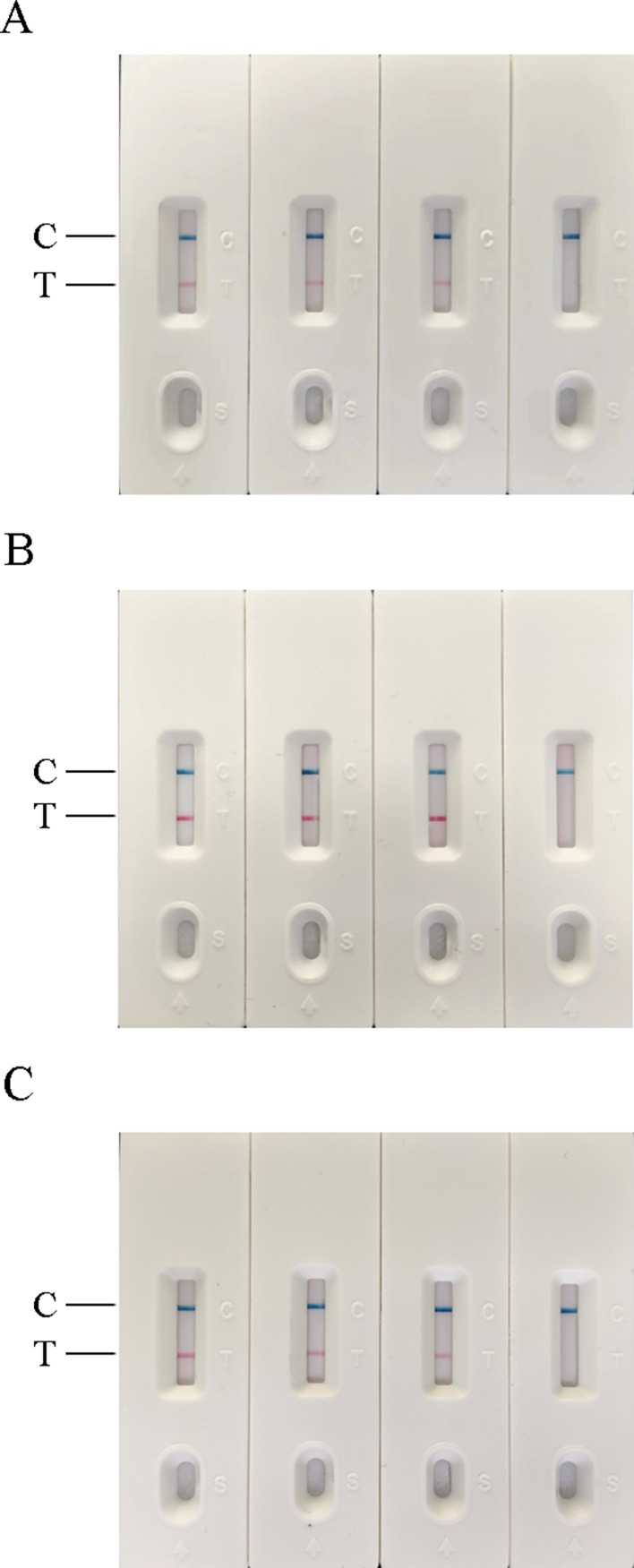
Repeatability and stability of RT-RAA-LFD method. (**A**), (**B**), and (**C**) in Fig. 11 were the repeatability and stability results of RT-RAA-LFD for H5, H7, and H9 subtypes of AIV, respectively. From left to right were three test groups and one negative control. In (**A**), (**B**), and (**C**), the results of the three test groups were positive and the results of the negative control were negative

The results of the RT-RAA-LFD method were shown in Fig. 11, three test groups showed positive and negative control showed negative. The results of the RF-RT-RAA method were shown in Table 1, and the coefficient of variation (CV) of the three test groups of AIV-H5, AIV-H7, and AIV-H9 were all less than 5%. Above results indicated that the two detection methods established in this experiment had good repeatability and stability.

**Table 1:** Repeatability and stability of RF-RT-RAA method

Virus	1 (min)	2 (min)	3 (min)	Mean (X ± SD)	CV (%)
AIV-H5	9.42	9.56	8.78	9.25 ± 0.42	4.54
AIV-H7	2.64	2.62	2.79	2.68 ± 0.09	3.36
AIV-H9	3.60	3.50	3.74	3.61 ± 0.12	3.32

### Clinical sample testing

RNA was extracted from 68 samples of sick chickens from farms with clinical symptoms that were suspected of AI, and AIV subtypes H5, H7, and H9 were detected, respectively. The results showed that only the AIV-H9 subtype was detected by RT-PCR-agarose electrophoresis, RFQ-RT-PCR, RT-RAA-LFD, and RF-RT-RAA. Based on RT-PCR-agarose electrophoresis, the clinical detection coincidence rate was 95.59% for RFQ-RT-PCR and RF-RT-RAA, 97.06% for RT-RAA-LFD (Table 2).

**Table 2:** The results of the four methods applied to the detection of clinical samples

Detection method	Number of positive H9 subtype	Negative number	Total coincidence rate*
RT-PCR	20	48	—
RFQ-RT-PCR	23	45	95.59%
RT-RAA-LFD	22	46	97.06%
RF-RT-RAA	23	45	95.59%

*Note* * represents the percentage of the number of both positive and negative samples in the total number of samples for the two test methods compared

## Discussion

AI has always been one of the major diseases plaguing the healthy development of the poultry industry. The outbreak of AI has brought huge economic losses to China and even the world, which can lead to human infection and death, threatening human health [28]. Therefore, the early and rapid detection of AIV is of great significance for its prevention and control.

It is common for different types of AIV to be caused by different HA subtypes. Therefore, this study aimed to establish RT-RAA-LFD and RF-RT-RAA for distinguishing H5, H7, and H9 subtypes of AIV. In this experiment, the HA gene was selected as the target sequence and DNAMAN software was used to analyze and compare the highly conserved and specific regions of each subtype sequence for designing primers and probes.

Different from other constant temperature detection methods, the RAA detection method only need a pair of preferred specific primers and probes, which is a new, rapid and sensitive in vitro isothermal nucleic acid amplification technology, the RT-RAA is based on RAA technology [29]. During the reaction, the compound, formed by the recombinase and the primers, finds the homologous sequence, following with a chain replacement reaction, which can rapidly amplify new DNA fragments in vitro after 15 ∼30 min reaction under isothermal conditions [30]. In this study, the RT-RAA-LFD method combined RT-RAA with LFD, using the nfo probe (46–52 bp) labeled with fluorescein (FAM) and forward/reverse primer (30–35 bp) labeled with biotin to realize the visualization of detection results. The probe has a tetrahydrofuran (THF) at least 30 bases away from the 5’ end and at least 15 bases away from the 3’ end, and the 3’ end is modified with a blocking group. The endonuclease does not work when the probe is single-stranded, but it can recognize and cleave THF when the probe binds to the homologous sequence to form a double-stranded amplification product, and finally forms a double-labeled amplification product with FAM and biotin. The detection results can be obtained within 3 min through LFD, realizing rapid and visual field detection [31]. The RF-RT-RAA method combines RT-RAA with exo probe (46–52 bp), which greatly improves the detection sensitivity and enables the detection of low-copy samples. Similarly, the THF site is labeled in the middle of the probe and required to be at least 30 bases away from the 5’ end and 15 bases away from the 3’ end. A fluorescent group and a quenching group were marked on both sides of the THF site respectively, and a blocking group was modified at the 3’ end. When the probe is single-stranded, the exonuclease does not work and the fluorescent signal emitted by the fluorescent group is absorbed by the quenching group. When the probe is combined with the homologous sequence to form a double-stranded amplification product, the exonuclease can recognize and cut THF, so that the signal emitted by the fluorescent group can be collected. With the extension of the amplification time, the fluorescent signal is continuously accumulated, which can be monitored in real time by the corresponding instrument.

Based on the above principle, RT-RAA technology was combined with test strip and fluorescent probe to establish two rapid detection methods for AIV in this experiment. In preliminary trials, to develop the two new methods for this study, multiple pairs of primers and probes were designed, and the best primers and probes were selected by agarose electrophoresis, based on the criteria of bright bands and conforming fragment size. The primers and probes used in this study were selected as optimal combinations. The results showed that the RT-RAA-LFD method can specifically amplify AIV-H5, AIV-H7, and AIV-H9 under the reaction conditions of 37 °C, 18 min, 39 °C, 20 min, and 38 °C, 18 min, respectively. The detection results can be directly observed by naked eyes within 3 min, which is more suitable for on-site detection. The RF-RT-RAA method can specifically amplify AIV-H5, AIV-H7, and AIV-H9 under the reaction conditions of 40 °C, 20 min, 38 °C, 16 min, and 39 °C, 17 min, respectively. It can monitor the amplification process and results in real time, which is more suitable for accurate laboratory diagnosis. Both RT-RAA-LFD and RF-RT-RAA established in this study have strong specificity and have no cross-reaction with pathogens with similar symptoms during the detection process. The detection has high sensitivity, the MDL of RT-RAA-LFD method is 10^2^ copies/µL, which is consistent with the sensitivity of RT-RAA-LFD method established by Wang et al. [32, 33], and is 10 ∼100 times higher than that of RT-PCR-agarose electrophoresis; the MDL of RF-RT-RAA method is 10^1^ copies/µL, which is slightly higher than the sensitivity of hepatitis B virus RAA method established by Shen et al. [34], and which is the same as that of African classical swine fever virus real-time fluorescence RAA method established by Zhao et al. [35] and severe acute respiratory syndrome coronavirus type 2 RF-RT-RAA method established by Wu et al. [36], which is 100 ∼1000 times higher than that of RT-PCR-agarose electrophoresis and consistent with the sensitivity of RFQ-RT-PCR method. In order to verify the stability of RT-RAA-LFD and RF-RT-RAA established in this study, we conducted a repeatability test, and the test results showed that the two methods had good repeatability and stability, the CV of RF-RT-RAA was less than 5%. In clinical application, both the coincidence rates between the two detection methods and ordinary agarose electrophoresis method are higher than 95%. In recent years, the main subtype of AIV prevalent in North China is H9, so only this subtype was detected in clinical samples. The results of this study were consistent with relevant literature reports [37, 38].

## Conclusion

In conclusion, based on RT-RAA technology, this study successfully established RT-RAA-LFD and RF-RT-RAA for AIV detection. Both two methods had the advantages of short detection time, simple operation, strong specificity, and high sensitivity, which were suitable for early, rapid, sensitive, and specific diagnosis of AIV in clinical field. RT-RAA-LFD was more suitable for grass-roots application with limited conditions, and RF-RT-RAA was more suitable for real-time monitoring in laboratories. The successful establishment of the two methods could provide help for AIV epidemiological investigation.

## Methods

### Nucleic acid extraction

The RNA of H5, H7, and H9 subtypes of AIV and Newcastle disease virus (NDV), as well as the cDNA of AIV-H1N1 (A/Hebei/F076/2018), AIV-H3N2 (A/Hebei/BD79/2018), Influenza B viruses (Victoria) (B/Hebei/CZ176/2018), and Influenza B viruses (Yamagata) (B/Hebei/HS639/2018) were preserved in the Animal Infectious Disease Laboratory of Hebei Agricultural University. Infectious bronchitis virus (IBV, AV1511) and avian infectious laryngotracheitis virus (ILTV, AV195) were purchased from China Veterinary Drug Administration. The viral DNA/RNA extraction kit (Tiangen Biochemical Technology Co., Ltd., Beijing, China) was used to extract the DNA or RNA of the sample for later use.

### Design of primers and probes

The hemagglutinin gene sequences of H5 (GenBank: CY014311.1), H7 (GenBank: MK453331.1), and H9 (GenBank: KP865956.1) subtypes were compared, respectively, and the conserved regions were selected. Primers and probes were designed according to the design principles of RT-RAA primers and probes. The preferred primers and probes were sent to the biotechnology company (Sangon Bioengineering Co., Ltd., Shanghai, China) for synthesis and labeling (Table 3).

**Table 3:** Design and modification of preferred primers and probes for H5, H7, and H9 subtypes of AIV

Detection method	Names	Sequence (5’→3’)	Gene location (bp)	Product size (bp)
RT-RAA-LFD	H5-LFD-F	AAGGCAATAGATGGAGTCACCAATAAGGTCAA	1165–1196	174
	H5-LFD-R	Biotin-AACCAGAAGTTCGGCATTATAAGTCCAAAC	1309–1338	
	H5-LFD-T	FAM-ATTTAATAACTTAGAAAGGAGAATAGAGAAT-THF-TAAACAAGAAGATGGA/C3-spacer/	1245–1292	
	H7-LFD-F	Biotin-TCAAATAACAGGGAAATTAAACCGGCTTATAG	1155–1186	193
	H7-LFD-R	CAGATCAATTGTATGCTGGTTCTCCATTGC	1318–1347	
	H7-LFD-T	FAM-ACAATGAATTCAATGAGGTAGAGAAGCAAAT-THF-GGTAATGTGATAAATTGG/C3-spacer/	1217–1266	
	H9-LFD-F	TCAGCGAGGTTGAAACTAGACTTAACATGATC	1223–1254	198
	H9-LFD-R	Biotin-CTTTCCCATCTTCCACCGCATTGGAACCCAATG	1388–1420	
	H9-LFD-T	FAM-CACTCGATGAGCATGATGCAAATGTAAACAATC-THF-ATATAATAAAGTGAAGA/C3-spacer/	1334–1384	
RF-RT-RAA	H5-RF-F	AAGGCAATAGATGGAGTCACCAATAAGGTCAA	1165–1196	174
	H5-RF-R	AACCAGAAGTTCGGCATTATAAGTCCAAAC	1309–1338	
	H5-RF-T	ATTTAATAACTTAGAAAGGAGAATAGAGAA/i6FAMdT//idSp//iBHQ1dT/AAACAAGAAGATGGA/C3-spacer/	1245–1292	
	H7-RF-F	TCAAATAACAGGGAAATTAAACCGGCTTATAG	1155–1186	193
	H7-RF-R	CAGATCAATTGTATGCTGGTTCTCCATTGC	1318–1347	
	H7-RF-T	ACAATGAATTCAATGAGGTAGAGAAGCAAA/i6FAMdT//idSp/GG/iBHQ1dT/AATGTGATAAATTGG/C3-spacer/	1217–1266	
	H9-RF-F	TCAGCGAGGTTGAAACTAGACTTAACATGATC	1223–1254	198
	H9-RF-R	CTTTCCCATCTTCCACCGCATTGGAACCCAATG	1388–1420	
	H9-RF-T	CACTCGATGAGCATGATGCAAATGTAAACAA/i6FAMdT/C/idSp/A/iBHQ1dT/ATAATAAAGTGAAGA/C3-spacer/	1334–1384	
RT-PCR	H5-F	TGGTAGATGGTTGGTATGGGTAC	1088–1110	316
	H5-R	ACCTTGTCGTAGAGGTTCTTAACA	1380–1403	
	H7-F	TCAAATAACAGGGAAATTAAACCG	1155–1178	193
	H7-R	CAGATCAATTGTATGCTGGTTCTC	1324–1347	
	H9-F	TCAGCGAGGTTGAAACTAGACTTA	1223–1246	198
	H9-R	CTTTCCCATCTTCCACCGCA	1402–1420	

/C3-spacer/: 3’ blocker; /i6FAMdT/: Fluorescent group; /iBHQ1dT/: Quench group; /idSp/: Gap of residues

### Establishment of RT-RAA-LFD reaction system

The reaction system was constructed according to the instructions of RT-RAA nucleic acid amplification kit (test strip method) (Qitian Gene Biotechnology Co., Ltd., Jiangsu, China): base buffer 25 µL, RT-RAA-LFD forward and reverse primers (10 µM) 2.1 µL, RT-RAA-LFD probe 0.6 µL, template 1 µL, purified water 16.7 µL, and finally 2.5 µL of magnesium acetate were added. The forward and reverse primers, probe, and template were corresponding to H5, H7, and H9 subtypes one by one, the same as below. The reaction tube was placed in the RAA-B6100 constant temperature oscillation mixer (Qitian Gene Biotechnology Co., Ltd., Jiangsu, China) for the reaction, the temperature was set to 39 °C and the time was 30 min. After the reaction, the nucleic acid amplification product was diluted 20 times with sterile ddH_2_O, thoroughly mixed, and 80 µL of diluted amplification product was dropped onto the sample hole of the test strip (Jiennuo Biomedical Technology Co., Ltd., Suzhou, China). After the quality control line (C) was colored, the test result of the test line (T) was read within 15 min.

Judgment standard: if the test strip only shows the C, the result is negative; if the test strip shows both the C and the T, the result is positive.

### Optimization of reaction conditions for RT-RAA-LFD detection method

The RT-RAA-LFD reaction temperature was set to 36, 37, 38, 39, 40, and 41 °C, respectively; the reaction was carried out according to the optimized temperature, and the reaction time was set to 14, 16, 18, 20, 22, 24, and 26 min, respectively; the reaction was carried out according to the optimized temperature and time, and the concentration gradients of primers and probes were 10,000, 5000, 2500, 1250, and 625 nmol/L, respectively. By comparing the reaction results, the color of the test line of the strip was observed. According to the single variable principle, the optimal reaction conditions could be judged by the following criteria: the lowest temperature, the lowest time, and the lowest primer and probe concentration required to achieve a clear detection line under different conditions.

### Establishment of RF-RT-RAA reaction system

A 50 µL reaction system was constructed according to the instructions of RT-RAA nucleic acid amplification kit (fluorescence method) (Qitian Gene Biotechnology Co., Ltd., Jiangsu, China): buffer 25 µL, RF-RT-RAA forward and reverse primers (10 µM) 2.1 µL each, RF-RT-RAA probe 0.6 µL, the remaining reagents and dosages were the same as RT-RAA-LFD. The primers and probes used for the H5, H7, and H9 detections were applied with the set of primers and probes corresponding to the names in Table 3, respectively, the same as below. The reaction tube with the added sample was placed in the pre-heated RAA-B6100 constant temperature oscillation mixer for pre-amplification. After that, the reaction tube was taken out and put into the constant temperature nucleic acid amplification detector RAA-F1620 (Qitian Gene Biotechnology Co., Ltd., Jiangsu, China). The reaction condition was set to 39 °C for 30 min, and the results were observed in real time.

Judgment standard: after the reaction, set the slope (k) to 20, and the instrument will automatically present the determination result. The result of k ≥ 20 of the fluorescence curve is positive.

### Optimization of reaction conditions for RF-RT-RAA detection method

The reaction temperatures of RF-RT-RAA were set at 37, 38, 39, 40, and 41 °C, respectively, and the reaction time was set at 30 min. The optimal reaction conditions were as follows: under different temperatures, the temperature with the highest fluorescence signal value was the optimal reaction temperature; only when the slope of the amplification curve reached the positive criterion (k ≥ 20) and its speed maintained stability, the optimal reaction time would present in the shortest time.

### Specificity test of RT-RAA-LFD and RF-RT-RAA for detection of AIV-H5, AIV-H7, and AIV-H9

According to the optimized reaction system, the RNA, cDNA or DNA of AIV-H5, AIV-H7, AIV-H9, AIV-H1N1, AIV-H3N2, Influenza B viruses (Victoria), Influenza B viruses (Yamagata), NDV, IBV, and ILTV was used as the template, and the forward and reverse primers and probes corresponding to H5, H7, and H9 subtypes were used to evaluate the specificity of RT-RAA-LFD and RF-RT-RAA.

### Construction of plasmid standard

Viral RNA was reverse transcribed into cDNA for PCR amplification in the following system: 2×Taq Mix 25 µL, cDNA template 2 µL, RT-PCR forward and reverse primers (10 µM) 1 µL each, purified water 21 µL filled to 50 µL, the forward and reverse primers and template according to the H5, H7, and H9 subtypes one by one. Reaction program: 94 °C for 5 min; denaturation at 94 °C for 30 s, annealing at 52 °C for 30 s, extension at 72 °C for 30 s, followed by 35 cycles of extension at 72 °C for 5 min, and storage at 4 °C. The gel was recovered by 2% agarose electrophoresis, purified and connected to the pMD20-T vector, then introduced into the competent cells. After the blue-white screening, suitable colonies were selected for overnight culture and plasmid extraction. The DNA copies number per unit volume of plasmid was calculated according to Moore’s law. The calculation formula is as follows: plasmid copy number (copies/µL) = [plasmid concentration (g/µL) × 6.02 × 10^23^] / [total fragment length (bp) × 660 g/mol], total fragment length = vector length (bp) + fragment length (bp).

The constructed standard plasmid was diluted to a gradient concentration of 10^9^-10^0^ copies/µL by 10-fold dilution method, and was stored at -20 °C for future use.

### Sensitivity test of four methods to detect AIV-H5, AIV-H7, and AIV-H9

The templates of RT-RAA-LFD method were 10^6^-10^0^ copies/µL plasmid in turn, and the reaction results were observed through the detection line of the test strip. The templates of RF-RT-RAA method were 10^4^-10^0^ copies/µL plasmid in turn, and the reaction results were observed by fluorescence signal. The templates of RT-PCR-agarose electrophoresis were 10^7^-10^0^ copies/µL of plasmid in turn, and 2% agarose electrophoresis was used to observe the results. The RFQ-RT-PCR reaction system included TB Green Premix Ex Taq™ II (TliRNaseH Plus) (2X) 12.5 µL, RT-PCR forward and reverse primers (10 µM) 1 µL each, template (10^6^-10^0^ copies/µL plasmid) 1 µL, filled with purified water to 25 µL, reaction procedure: 95 °C for 30s; the results were observed after 40 cycles at 95 °C for 5 s and 60 °C for 30 s. Negative water control was set for the above tests.

### Repeatability and stability test of RT-RAA-LFD and RF-RT-RAA for detection of AIV-H5, AIV-H7, and AIV-H9

In order to verify the repeatability and stability of the two detection methods, the same concentration of AIV-H5, AIV-H7, and AIV-H9 plasmids were used as templates (10^2^ copies/µL for RT-RAA-LFD and 10^4^ copies/µL for RF-RT-RAA), and each was repeated by three times, and negative control was set. The optimized RT-RAA-LFD method and RF-RT-RAA method were used for the test. The time recorded when the fluorescence curve reached the positive standard and the CV was calculated.

### Detection of clinical samples

The clinical samples were collected from several individual chicken farms in northern China, and the animals were released after collecting throat swabs. The sick birds mainly presented with mild or severe respiratory symptoms, consistent with the clinical symptoms of AI. Sixty-eight throat swabs were collected and detected by RT-PCR-agarose electrophoresis, RFQ-RT-PCR, RT-RAA-LFD, and RF-RT-RAA, respectively. The coincidence rates of several detection methods were compared based on RT-PCR-agarose electrophoresis.

### Electronic supplementary material

Below is the link to the electronic supplementary material.

Supplementary Material 1

Supplementary Material 2

Supplementary Material 3

## Data Availability

The dataset analyzed during the current study is available from the corresponding author on reasonable request.

## Abbreviations

AI: Avian influenza
AIV: Avian influenza virus
WOAH: World Organization for Animal Health
RT-PCR: Reverse transcription polymerase chain reaction
RFQ-RT-PCR: Real-time fluorescence quantification reverse transcription polymerase chain reaction
RPA: Recombinase polymerase amplification
RAA: Recombinase-aided amplification
PCR: Polymerase chain reaction
LFD: Lateral flow dipstick
HA: Hemagglutinin
RT-RAA: Reverse transcription recombinase-aided amplification
RT-RAA-LFD: Reverse transcription recombinase-aided amplification lateral flow dipstick
RF-RT-RAA: Real-time fluorescence reverse transcription recombinase-aided amplification
MDL: Minimum detection limit
FAM: Fluorescein
THF: Tetrahydrofuran
CV: Coefficient of variation
NDV: Newcastle disease virus
IBV: Infectious bronchitis virus
ILTV: Avian infectious laryngotracheitis virus
C: Control line
T: Test line
RF: Real-time fluorescence
