# Supplementary figures and images for: Detection method for reverse transcription recombinase-aided amplification of avian influenza virus subtypes H5, H7, and H9
Source: BMC Vet Res. 2024 May 16;20:203. doi: 10.1186/s12917-024-04040-9 (PMC11097555; doi:10.1186/s12917-024-04040-9)

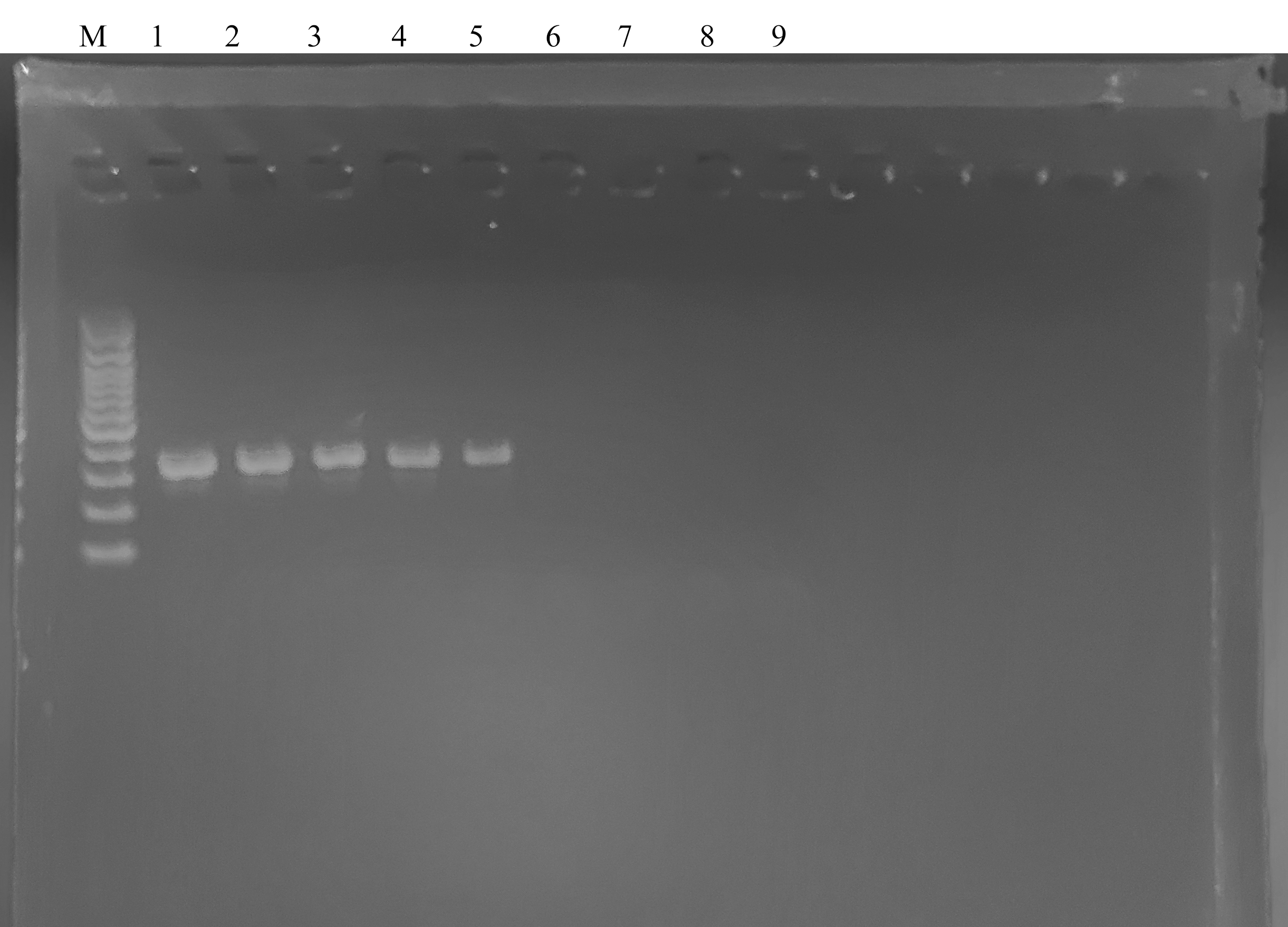

Supplement: Supplementary file 1 — Supplementary Material 1 [file 12917_2024_4040_MOESM1_ESM.tif]

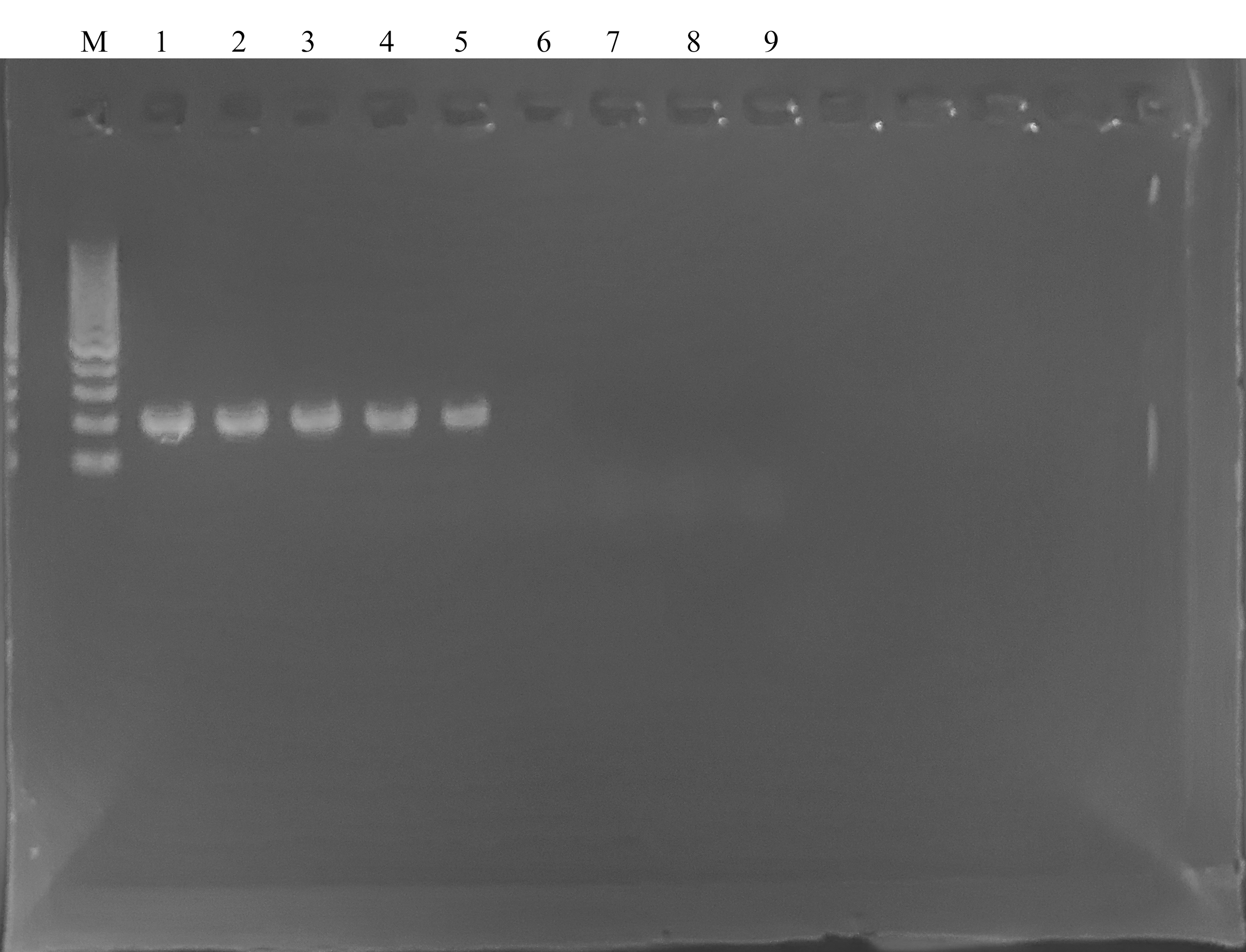

Supplement: Supplementary file 2 — Supplementary Material 2 [file 12917_2024_4040_MOESM2_ESM.tif]

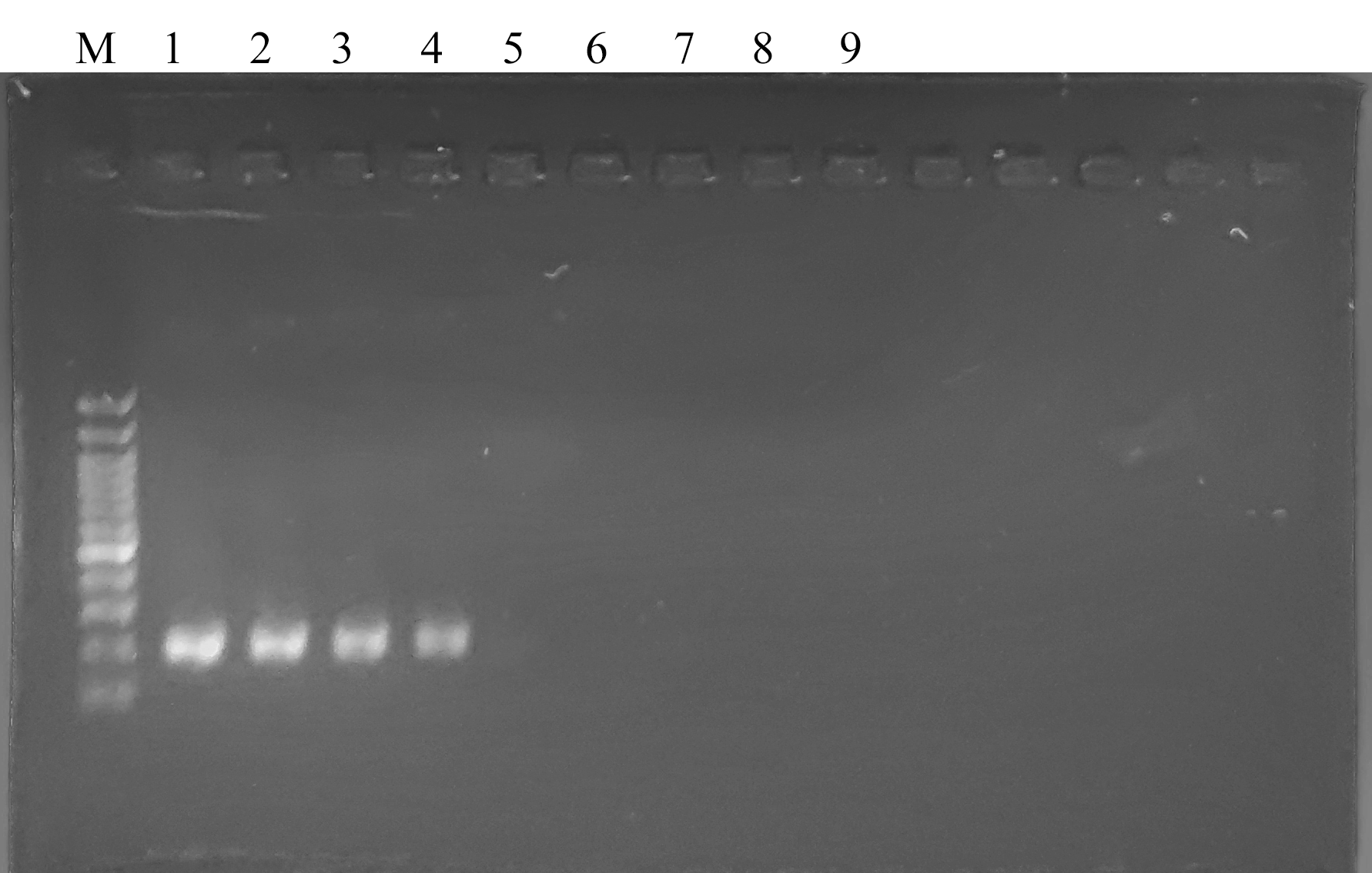

Supplement: Supplementary file 3 — Supplementary Material 3 [file 12917_2024_4040_MOESM3_ESM.tif]
